# Fewer bowl traps and more hand netting can increase effective number of bee species and reduce excessive captures

**DOI:** 10.1002/ece3.11036

**Published:** 2024-02-26

**Authors:** Diane L. Larson, Nora Pennarola, Julia B. Leone, Jennifer L. Larson

**Affiliations:** ^1^ U.S. Geological Survey, Northern Prairie Wildlife Research Center St. Paul Minnesota USA; ^2^ U.S. Fish and Wildlife Service Bloomington Minnesota USA; ^3^ Friends of the Mississippi River St. Paul Minnesota USA; ^4^ USDA Forest Service, Forest Health Protection St. Paul Minnesota USA

**Keywords:** bee survey methods, bee‐habitat associations, Hill numbers, sampling effort

## Abstract

Reports increasingly point to substantial declines in wild bee abundance and diversity, yet there is uncertainty about how best to measure these attributes in wild bee populations. Two commonly used methods are passive trapping with bee bowls or active netting of bees on flowers, but each of these has drawbacks. Comparing the outcomes of the two methods is complicated by their uncomparable units of effort. The abundance distribution of bee species is also typically highly skewed, making it difficult to accurately assess diversity when rarer species are unlikely to be caught. The effective number of species, or Hill numbers, provides a way forward by basing the response metric on the number of equally abundant species. Our goal is to compare the effective number of bee species captured between hand netting and bowl trapping in wheatgrass prairie in South Dakota and tallgrass prairie in Minnesota, USA. Species overlap between the two methods ranged from ~40% to ~60%. Emphasis placed on rare species was important, so that 95% confidence limits overlapped between the two methods for species richness but netting exceeded trapping for Shannon's and Simpson's diversities. Netting always captured more bee species with fewer bee individuals than trapping. In most cases, the number of bees captured in bowl traps indicated substantial over‐sampling, with little increase in bee species detected. Correlations between bee and floral abundance, richness, and diversity differed between netted and trapped samples. We conclude that netting and trapping together produce a more complete account of species richness, but shifting sampling emphasis from trapping to netting will result in fewer bees, but more bee species captured. Due to the different relationships between bee and floral diversities that depended on sampling method, it is unwise to compare habitat associations determined by netting with those determined by trapping.

## Graphical abstract

Reports increasingly point to substantial declines in wild bee abundance and diversity, yet there is uncertainty about how best to measure these attributes in wild bee populations. We compared two common survey techniques, trapping using cups filled with soapy water and targeted netting. Although both methods are needed to obtain the most complete species list, shifting sampling emphasis from trapping to netting will result in fewer bees, but more bee species captured.

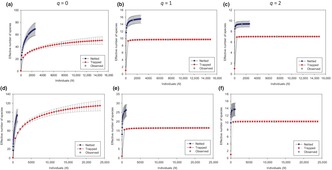

## 1 INTRODUCTION

The importance of having robust statistical data on bee distribution and abundance cannot be overstated. As we gather more information, the evidence supporting the possibility of an ongoing “insect apocalypse” becomes increasingly compelling (Goulson, 2019; Wagner, 2020). Pollinator declines have long been a subject of concern (Potts et al., 2010). Among pollinating insects, bees are essential in both agricultural settings (Reilly et al., 2020) and natural systems (Spira, 2001) and declines in abundance and richness have been documented in several studies (Bartomeus et al., 2013; Cameron et al., 2011; Grixti et al., 2009; Thomson, 2016; Turley et al., 2022; Zattara & Aizen, 2021). Even if we accept that the preponderance of evidence indicates declines, the data required to devise effective management plans may still be lacking (Chesshire et al., 2023; MacIvor & Packer, 2016). Detection probabilities for different species using common sampling methods (e.g., various passive trapping methods and/or focused or sweep netting) are unknown, which reduces inferences that can be made about the extent of the declines (Didham et al., 2020; Portman et al., 2020).

Methods used to obtain distribution and abundance data for wild bees are limited, and each has drawbacks (O'Connor et al., 2019). Bowl traps (also known as bee bowls or pan traps) have been recommended as one of the more unbiased methods to obtain data about bee species inhabiting an area of interest (Westphal et al., 2008). It should be noted that “unbiased” refers to biases associated with human expertise in deploying the traps, not with taxonomic bias, of which bowl traps are not exempt. Reports of capture results for bowl traps are heavily weighted toward the Halictidae (Cane et al., 2000; Droege et al., 2010; Grundel, Frohnapple, et al., 2011; Westphal et al., 2008). In addition, captures of non‐target insect species, or bycatch, can be substantial in passive trapping methods (Montero‐Castano et al., 2022). While the link between captures from bowl traps and the overall population abundance of bee species remains uncertain, these traps serve as valuable tools for recording the distribution of the captured bee species (Packer & Darla‐West, 2021). There is evidence that capture rates in bowl traps are depressed when floral resources are abundant (Baum & Wallen, 2011; Cane et al., 2000). Biases related to bowl color, size, and relationship to vegetation height have been discussed, as well (Packer & Darla‐West, 2021). For example, Grundel, Jean, et al. (2011) found preferences for different bowl colors among seasons and habitat types. Packer and Darla‐West (2021) summarized what little information exists on bowl size (concluding that larger vessels capture more bees, but fewer per volume of liquid) and relationship to vegetation height (traps hidden in vegetation will not be seen by bees).

Hand netting bees on flowers, in contrast to bowl traps, requires field personnel who are skilled in the use of nets and adept at detecting bees of different sizes that land on flowers. Parasitic bees that spend less time at flowers are often underrepresented in netted samples and netting may be biased toward larger bees that are more easily observed (Westphal et al., 2008). While bowl traps can be left out for 24 h or more, netting is limited to the amount of time personnel can spend in the field under conditions appropriate for bees to be foraging. As with bowl traps, there is no clear relationship between captures and population‐level abundance (Kuhlman et al., 2021). Hand netting allows ecological inferences of the bees' relationships to floral resources if flower species are recorded as bees are captured, whereas trapping may capture dispersing bees with no relationship to the habitat (Packer & Darla‐West, 2021).

Both bowl traps and hand netting are often used in either inventory (Meiners et al., 2019) or hypothesis‐driven research (Lasway et al., 2022; Leone et al., 2022b; Watson et al., 2011) because each method will capture somewhat different species. Common species have been found to be equally well represented by both methods, but rarer or more specialized species may be best captured by only one of the methods (Grundel, Frohnapple, et al., 2011). Several studies have made comparisons between these, and other, bee survey methods (Campbell et al., 2023; Krahner et al., 2021; Pei et al., 2022), but comparisons are hampered by uncomparable sampling effort: How does one compare bowls deployed for a number of hours, awaiting discovery by bees, with intensive sampling of bees that are in the act of foraging? Even if the number of hours each method is deployed is the same, sampling effort may not be equivalent in any meaningful way. Moreover, because the number of species in a sample generally increases with the number of individuals captured, comparisons based on equal hours deployed may favor techniques that capture more individuals/unit time, despite ultimately comprising fewer species when capture numbers are standardized (Roswell et al., 2021). This is an important point to consider as we strive to minimize the number of bees killed during sampling.

The species abundance distribution of bee communities can strongly influence estimates of species diversity (Roswell et al., 2021). If all species were equally common and equally detectable, the likelihood of sampling any of the species would be similar, at least given a single sampling technique; the reality is that bee communities often include a substantial number of rare or uncommonly captured species, and different sampling methods will not capture all species with equal probability. This seems especially relevant when comparing species richness estimates based on bowl traps versus hand netting, as described above. In light of concerns about declining bee numbers and diversity, it is important to use methods that both achieve the goals of a study in terms of sample completeness and minimize the number of destructively sampled bees.

Given these considerations, we base our comparisons of netted and trapped bee diversity on the effective number of species, also known as Hill numbers (Chao, Chiu, & Jost, 2014), as recommended by Roswell et al. (2021). Effective number of species expresses diversity as the number of *equally abundant species*, which allows direct comparison of diversity measures among methods (Chao, Chiu, & Jost, 2014; Chao, Gotelli, et al., 2014). Diversity order (*q*) specifies the sensitivity of the measure to the relative frequencies of the species; as *q* increases from 0 (equivalent to species richness) through 1 (equivalent to Shannon diversity) and 2 (equivalent to Simpson diversity), sensitivity to rare species declines (Hsieh et al., 2016). This allows us to examine how the emphasis on rare (*q* = 0), common (*q* = 1), or dominant (*q* = 2) species affects the relative performance of netting versus trapping. We also compare the effect of number of captured individuals on effective number of species in netted and trapped samples. In contrast to effort, the number of captures allows comparison based on a common metric and provides an indication of how many bees each method must sample (which for bees typically involves killing them to allow identification) to achieve a desired level of sample completeness (i.e., coverage) in the habitats studied.

Our overall goal is to compare bee species captured, in terms of effective number of species at various values of *q*, between targeted netting (i.e., netting restricted to those bees interacting with the reproductive structures of flowers; hereafter “hand netting” or simply “netting”) and standardized bowl traps deployed for 24 h (hereafter “bowl traps” or simply “traps”). We ask: (1) Do methods vary in their characterization of the effective number of species depending on the importance of rare species in the survey? (2) At what number of individuals captured is diversity no longer increasing and does this vary between the two methods? (3) Do the two methods vary in efficacy among bee families; and (4) Is there a correlation between bee and flower or forb abundance, species richness, evenness, Shannon or Simpson diversity derived from the two bee sampling methods?

## 2 METHODS

We use two data sets from different habitats in which both bowl traps and hand netting were used to assess relative abundance of bee species; data were not explicitly collected to test differences in the two methods but lend themselves well to this application. Both sampling methods were deployed at the same time at each of several study sites. Netting was done by the same person or group of people during optimal conditions for bee captures, approximately 10 am to 5 pm. One data set is from a study that took place at Badlands National Park, South Dakota, USA, in wheatgrass prairie during June and July of 2012 (hereafter, wheatgrass sites). Details can be found in Larson et al. (2016). Briefly, the study encompassed 12 1‐ha plots, each of which contained 10 2‐m × 75‐m transects. Within each transect, all insects in contact with reproductive parts of flowers were netted during 20‐min time‐constrained surveys (total time per plot per visit was 200 min) by one of six observers, half of whom had >2 years of experience netting bees; observers rotated through the plots, so all plots were sampled by all observers at least once. Plots were sampled with nets every‐other day during each of two sample periods (June 22–30 and July 8–17) for a total of 87 plot samples. Thirty white, fluorescent blue, and fluorescent yellow 3.25‐oz (96 mL) plastic bowls half‐filled with soapy water were placed on the ground at approximately 5‐m intervals on a diagonal across each plot after netting on most days and retrieved about 24 h later. There were 78 trapped plot samples. Flower counts were conducted once or twice at each plot within each sample period along the same transects used for bee netting (see Larson et al. (2016) for details).

A second study had 20 plots of varying sizes on remnant tallgrass prairies in western Minnesota, USA (hereafter tallgrass sites). Detailed methods can be found in Leone et al. (2022b). The 20 study sites were located in the prairie parkland region of western Minnesota and ranged in size from 1.13 to 144.7 ha. Bees were netted by one experienced graduate student observer during time‐constrained meandering walks, with the time proportional to the size of the site, within a range of 30–120 min. Netting was intended as a way to acquire a more complete species list than could be obtained by bowl trapping alone. Bowl traps (30 3.25‐oz [96 mL] white, fluorescent yellow, and fluorescent blue plastic bowls half‐filled with soapy water) were placed in groups of three, elevated to vegetation height at 20‐m intervals along permanently marked transects at each site, and left in place for 24 h. Sites were visited three times per year between June 15 and August 31, 2016, and May 14 and August 18, 2017; hand netting and bowl trapping were conducted concurrently during each visit, with netting performed between 10 am and 5 pm. Forb frequency was determined once at each site each year using methods described in Larson et al. (2020). Bee data were pooled across years and sites for all analyses except to evaluate association with forb frequency and diversity, for which site‐level data were retained.

### 2.1 Data analysis

Data were analyzed using R version 4.2.1 (R Core Team, 2022) unless otherwise noted. We calculated Hill diversity (effective number of species; Chao and Jost (2015)) distributions for *q* = 0–3 for netted and trapped samples at each of the two sites using the Diversity function in the SpadeR package (Chao et al., 2016). Total captures per species were summed over the length of each study (2 months or 2 years for wheatgrass and tallgrass, respectively). We used the ChaoShared function in SpadeR to estimate the number of shared species in the netted and trapped samples at each site and report the abundance‐based coverage estimator for heterogeneous samples, which allows for heterogeneous discovery probabilities. Significant differences were assumed between netted and trapped estimates if 95% confidence intervals, calculated from 50 bootstrap replicates, did not overlap.

We used the iNext package (Hsieh et al., 2016) to calculate effective number of species for *q* = 0, 1, and 2 as a function of number of individuals captured. Data were summarized as above; we report the effective number of species at the observed number of captures as well as rarefied (to 1 capture) and extrapolated (to double the number of observed captures) for netted and trapped samples at each site. Similarly, total captures per species were summarized by bee family and iNext was used to calculate effective number of species at *q* = 0, 1 and 2 as a function of number of individuals of each family captured by each method at each site. We used SpadeR to calculate coverage for each bee family by method and site. Significant differences between netted and trapped samples were assumed if 95% confidence intervals, calculated from 50 bootstrap replicates, did not overlap.

Flower counts (at wheatgrass sites) and forb frequency (at tallgrass sites) per plot per sample period were entered into species‐by‐plot/period matrices, and abundance, species richness, evenness, and Shannon and Simpson diversities were calculated using the Summary function in PCOrd version 7 (McCune & Mefford, 2018). Similarly, bee species‐by‐plot/period matrices were used to obtain abundance, species richness, evenness, and Shannon and Simpson diversities using the Summary function in PCOrd. We calculated correlation coefficients between floral and bee abundance and diversity measures with proc corr in SAS version 9.4 ((c) 2016 by SAS Institute Inc., Cary, NC, USA).

## 3 RESULTS

**FIGURE 1:**
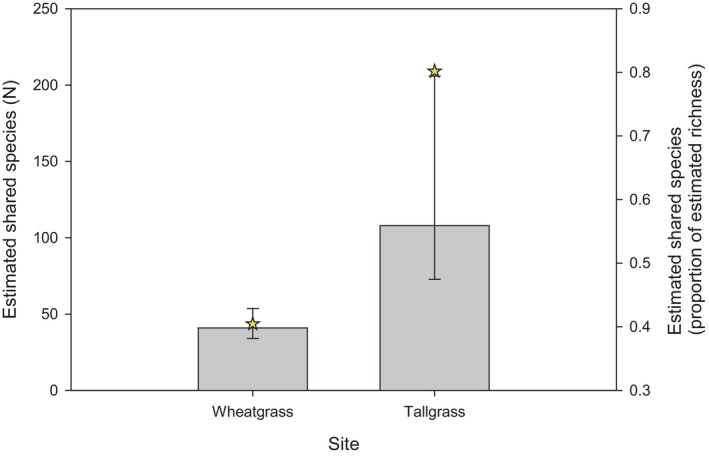
Overlap in bee species captured by netting and trapping. Bars indicate estimated number of shared species for each site and 95% confidence intervals; stars indicate shared species as a proportion of total estimated richness for each site.

In wheatgrass sites, 15% of individuals captured were caught in nets, which included representatives of 83% of the total species captured by both methods combined; trapping captured 85% of individuals and 63% of the species (Table 1). In tallgrass sites, 4.6% of individuals and 59% of total species were captured in nets; 95% of individuals and 86% of all species were captured in traps (Table 1). Only 40% of species were shared between netted and trapped collections at wheatgrass sites, while 80% of species were shared between the two methods at tallgrass sites (Figure 1 and Tables A1 and A2). Chao‐Jost estimates of species richness were ~42% higher than observed in wheatgrass sites and ~13% higher in tallgrass sites (Table 1).

**TABLE 1:** Summary statistics for hand‐netted, bowl‐trapped, and combined (netted + trapped) samples of bee species at four study sites in wheatgrass prairie in South Dakota and tallgrass prairie in Minnesota, USA, in June and July 2012.

Site	Method	Sample size (*n*)	Species (*N*)	Chao‐Jost richness	Shared species (CV)
Wheatgrass	Netted	1303	59		
	Trapped	7418	45		
	Combined	8721	71	101.080	40.881 (9.449)
Tallgrass	Netted	572	70		
	Trapped	11,901	102		
	Combined	12,473	119	134.624	107.925 (30.756)

Abbreviation: CV, coefficient of variation.

**FIGURE 2:**
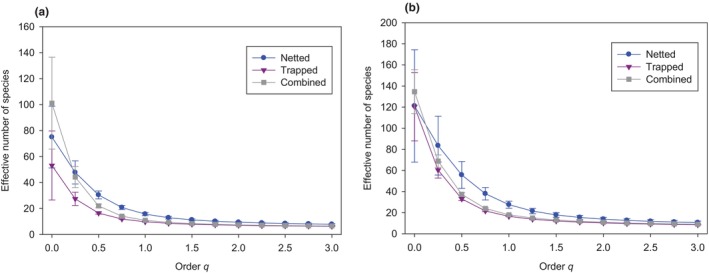
Hill diversity estimates over *q* = 0 to *q* = 3 at (a) wheatgrass prairie in South Dakota and (b) tallgrass prairie in Minnesota for netted, trapped, and netted and trapped bee species combined. Shown are Chao‐Jost estimates of effective number of species (Hill numbers) and 95% confidence limits. As diversity order (*q*) increases from 0 to 3, sensitivity to rare species declines.

Species diversity distributions: Trapping was never the best strategy to maximize estimates of effective number of species (Hill numbers) at any level of *q* (Figure 2). At *q* = 0, where every species is equally weighted, netting and trapping confidence intervals overlapped at both sites. Combining the methods increased mean effective number of species at *q* = 0, but still fell within 95% confidence intervals of netted or trapped values. Netting better captured the diversity of abundant species (*q* = 1); netted diversity of abundant species was 54% and 61% higher than trapped at wheatgrass and tallgrass sites, respectively. At *q* = 2 and beyond, netted and trapped were equivalent at the two sites, meaning that dominant species were similarly likely to be captured by netting or trapping.

**FIGURE 3:**
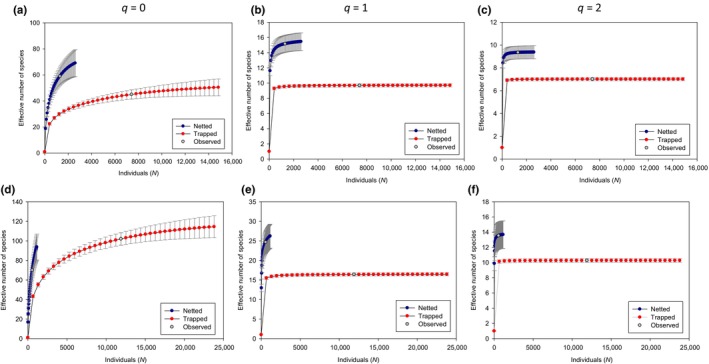
Effective number of species at *q* = 0, 1 or 2 as a function of number of individuals collected in netted and trapped samples in wheatgrass prairie in South Dakota (a–c) and tallgrass prairie in Minnesota (d–f). Plotted values below the observed point were obtained via rarefaction and those above the observed point by extrapolation. Note that scale of *Y*‐axes varies. As diversity order (*q*) increases from 0 to 2, sensitivity to rare species declines.

Diversity as a function of number of captures: At both sites and at all levels of *q*, netting captured a higher number of species as a function of number of individual captures and confidence intervals did not overlap with trapping estimates (Figure 3). Effective number of species for trapped samples leveled off well below observed capture numbers for both *q* = 1 and *q* = 2, reflecting a substantial amount of trapping that produced no new abundant or dominant species. For example, in wheatgrass sites, the effective number of species in bowl traps at *q* = 1 increased by only 0.39 effective species between 413 captured individuals as the curve leveled off and 7418 individuals when trapping ceased. On the other hand, netted diversity was still increasing in tallgrass, but not at wheatgrass sites, at *q* = 1 and 2. When netting ceased in tallgrass sites, 572 individual bees yielded 24.7 effective species; at the same sites, when trapping ceased, 11,901 individuals yielded 16.4 effective species. At *q* = 0, neither method reached an asymptote at either site.

**FIGURE 4:**
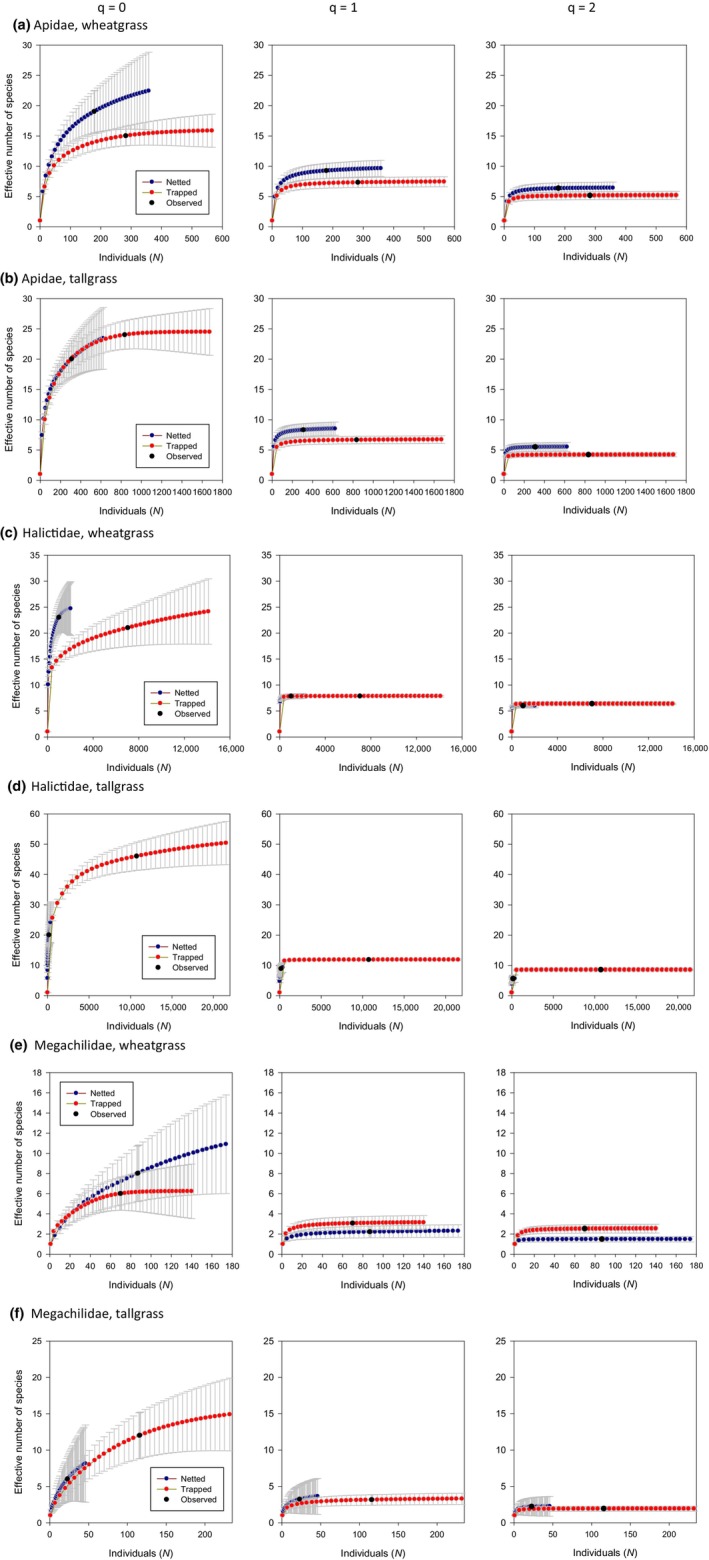
Effective number of species at *q* = 0, 1 and 2 by bee family as a function of number of individuals collected for netted and trapped samples in wheatgrass prairie in South Dakota and tallgrass prairie in Minnesota, USA. Apidae in wheatgrass (a) and tallgrass (b); Halictidae in wheatgrass prairie (c) and tallgrass (d); and Megachilidae in wheatgrass (e) and tallgrass (f). Plotted values below the observed point were obtained via rarefaction and those above the observed point by extrapolation. As diversity order (*q*) increases from 0 to 2, sensitivitry to rare species declines.

**FIGURE 5:**
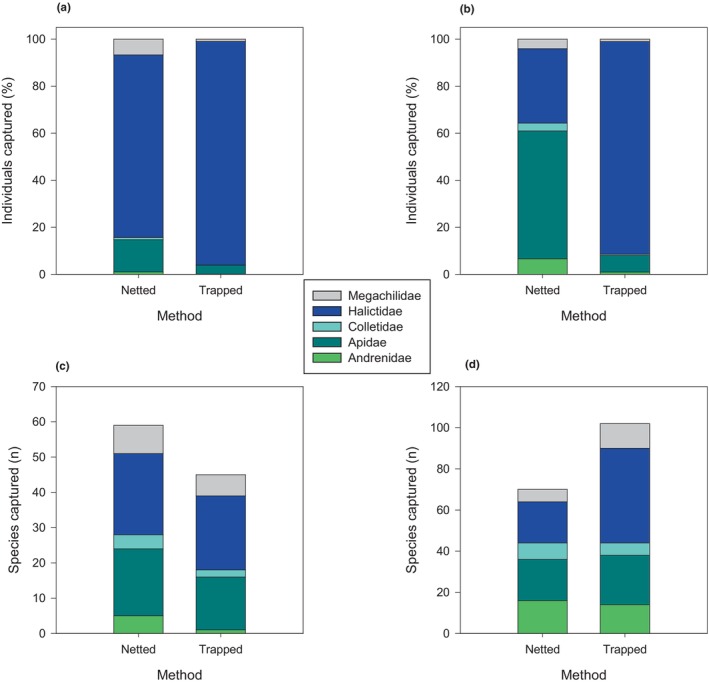
Stacked graphs showing bee families as a percentage of total captures at (a) wheatgrass sites in South Dakota and (b) tallgrass sites in Minnesota, USA, and number of species captured per bee family at (c) wheatgrass and (d) tallgrass sites, using hand nets or pan traps. Data presented are based on raw counts of species and individuals.

Diversity within bee families: Only once, for megachilids in wheatgrass sites at *q* = 2, was there a clear advantage to trapping over netting: Trapping captured a greater diversity of dominant megachilids than did netting. In most cases, confidence limits overlapped (Figure 4), although too few halictids were captured by nets in tallgrass prairie to be sure of the trajectory of the curve. In wheatgrass and tallgrass sites, >90% of trapped captures were halictids (Figure 5 and Tables A1 and A2). Halictids were also captured with high frequency (78% of total captures) in netted samples in wheatgrass sites, but at much lower frequencies (33% of total netted captures) at tallgrass sites. In raw numbers, more species were captured in bowl traps than by netting in tallgrass sites (Figure 5d), but this came at the expense of an extra 11,329 individual bees killed.

Relationships between floral resources and bees: At wheatgrass sites, trapped bee species richness and Shannon diversity were negatively correlated with floral diversity (Table 2). None of the netted bee metrics were correlated with floral metrics at wheatgrass sites. In contrast, at tallgrass sites, forb frequency, species richness, and Shannon's diversity all had significant positive correlations with netted bee abundance; netted bee richness was also positively correlated with forb abundance and richness, but forb frequency was negatively correlated with netted bee evenness (Table 2). Trapped bees were not correlated with any of the floral metrics at tallgrass sites. None of the correlations were strong at either site.

**TABLE 2:** Correlation coefficients and their *p*‐values between floral resource abundance (measured as flower counts in wheatgrass sites (*n* = 24) and forb frequencies in tallgrass sites (*n* = 20)), species richness, evenness, Shannon diversity (*H*′), and Simpson diversity (*D*); and abundance, richness, evenness, and Shannon and Simpson diversities of netted and trapped bees at wheatgrass and tallgrass sites in South Dakota and Minnesota, respectively.

Site	Floral resource	Netted bees		Evenness	*H*′	*D*	Trapped bees		Evenness	*H*′	*D*
		Abundance	Richness				Abundance	Richness			
Wheatgrass	Flower count	−.078	.005	.010	.052	.037	−.058	−.174	.162	.012	.063
	*p*	.717	.982	.964	.810	.864	.789	.417	.450	.954	.771
	Flower richness	−.209	−.229	−.014	−.221	−.158	−.098	**−.506**	−.093	**−.427**	−.305
	*p*	.328	.281	.948	.298	.462	.649	**.012**	.665	**.038**	.147
	Flower evenness	−.212	−.183	.147	−.096	−.012	−.013	.136	−.137	−.043	−.066
	*p*	.320	.393	.492	.654	.954	.952	.526	.522	.844	.761
	Flower *H*′	−.206	−.151	.050	−.091	−.049	−.054	−.084	−.061	−.140	−.122
	*p*	.333	.480	.815	.671	.819	.801	.698	.778	.514	.572
	Flower *D*	−.236	−.165	.069	−.091	−.054	−.035	−.040	−.066	−.116	−.110
	*p*	.266	.441	.749	.674	.802	.871	.853	.758	.589	.609
Tallgrass	Forb frequency	**.574**	**.630**	**−.461**	.296	−.076	.131	.006	.018	.016	.051
	*p*	**.008**	**.003**	**.041**	.205	.751	.583	.980	.941	.946	.830
	Forb richness	**.593**	**.501**	−.442	.244	−.047	.385	−.089	−.302	−.270	−.251
	*p*	**.006**	**.025**	.051	.301	.845	.094	.710	.196	.250	.285
	Forb evenness	−.014	−.257	−.086	−.259	−.145	.144	.192	.087	.156	.122
	*p*	.954	.274	.720	.270	.542	.546	.417	.715	.511	.607
	Forb *H*′	**.479**	.317	−.419	.098	−.110	.395	−.026	−.221	−.181	−.178
	*p*	**.033**	.173	.066	.682	.644	.085	.915	.350	.446	.452
	Forb *D*	.332	.220	−.392	.006	−.163	.266	.033	−.029	−.008	−.013
	*p*	.153	.351	.087	.982	.493	.258	.891	.904	.974	.955

*Note*: Numbers in bold indicate statistical significance (*p* < .05).

## 4 DISCUSSION

The goal of this study was to compare netting and trapping in ways that did not rely on uncomparable measures of effort. In virtually every comparison we made, netting captured as many or more effective numbers of species than trapping. The advantage of netting over trapping was most obvious when comparing total effective number of species observed as a function of number of individual bees captured: in no case did trapping surpass netting at equivalent number of individuals captured. Species richness (*q* = 0) was poorly estimated by both netting and trapping, but netting and trapping results converged for dominant species (*q* > 1.5). Estimated number of shared species between netting and trapping, which was ≤60%, indicates that both methods will be needed for the most complete species inventory, as many others have noted (Grundel, Frohnapple, et al., 2011; Minckley & Radke, 2021; Roulston et al., 2007; Wilson et al., 2008). Nonetheless, the effective number of species when netted and trapped captures were combined declined rapidly as *q* increased and was less than netting alone between *q* = 0.5 and *q* = 1.5. Trapping therefore may add little to estimates if the goals of the study can be achieved with Shannon diversity rather than species richness.

Differences between effective numbers of species collected by netting or trapping were sensitive to the emphasis placed on species' relative abundances. At *q* = 0 (species richness), where all species are considered without regard to their relative abundances, confidence intervals were large and overlapped substantially. Others have cautioned that species richness is a poor measure due to its reliance on detecting rare species (Roswell et al., 2021) and our results parallel this observation. Grundel, Frohnapple, et al. (2011) noted that both netting and trapping will capture the common species. We also found this to be true, in that netting and trapping had similar effective numbers of species at *q* > 1.5.

Comparisons between effective number of species detected by netting versus trapping as a function of number of individual bees captured indicate how important it is for investigators to clearly define their objectives. If Shannon or Simpson diversities are appropriate response metrics, our results indicate substantial over‐sampling at our study sites, especially with traps. At *q* = 1 (abundant species, Shannon diversity), effective number of species was still increasing for netted samples at the observed number of individuals captured, but it had leveled off with far fewer captures for trapped samples. The difference was even more stark for *q* = 2 (dominant species, Simpson diversity). In these cases, redirecting effort from trapping to netting would likely increase the effective number of species detected. Even at *q* = 0, netting produced greater effective numbers of species than trapping for a given number of individual bees captured, arguing for the value of rebalancing effort toward netting. In addition to improving efficiency, reducing the total number of bees captured can be considered a best practice for biodiversity research (Montero‐Castano et al., 2022), keeping in mind that bees—with the exception of bumble bees—are nearly always destructively sampled.

A few studies have used the number of individuals captured to standardize species richness. Grundel, Frohnapple, et al. (2011) also found that netting detected more species than bowl traps for a given number of individual bees captured, and O'Connor et al. (2019) found netting to capture more bumble bee species but trapping to capture more solitary bees. Most studies have used other means of standardization to compare netted and trapped bee metrics. In urban areas around Perth, Australia, “target sweep netting” resulted in more total captures and more taxonomic units captured than concurrent pan trapping, using nine 350‐mL yellow and 20 96‐mL yellow or blue bowls, when each method was deployed for the same number of hours (Prendergast et al., 2020). Roulston et al. (2007) also found greater numbers and richness of bees captured with nets (13.5 person‐hours) than with pan traps (30 traps deployed for 9 h) on the same 1‐ha site. In contrast, Westphal et al. (2008) found pan traps (6 deployments of 15 traps for 48 h) to outperform netting (10 50‐min standardized transect walks) on transects across a variety of seminatural and agricultural sites across Europe. As argued above, it is unclear how comparable these measures of effort are, but regardless of how comparisons are made, there is little consensus on whether netting or trapping captures more species. Nonetheless, it is clear that using both methods simultaneously will produce the most complete species list (i.e., species richness; Grundel, Frohnapple, et al., 2011; Lasway et al., 2022; Roulston et al., 2007; Wilson et al., 2008).

Many studies have reported that halictids are by far the most common family captured in bowl traps (Cane et al., 2000; Droege et al., 2010; Grundel, Frohnapple, et al., 2011; Westphal et al., 2008). This was certainly the case at our sites, in each of which >90% of trapped captures were halictids. Portman et al. (2020) argued that halictids, due to both difficulty in identification and their association with weedy habitats, are a poor choice for monitoring and thus reduce the value of bowl traps by their extreme taxonomic bias toward this family. The probability of misidentifications can be high in the more difficult groups within Halictidae, rendering conclusions about distributions and trends suspect (Portman & Tepedino, 2021). Indeed, Chesshire et al. (2023) found higher taxonomic richness among halictids in the eastern than western United States, but considered that taxonomic ambiguities may have resulted in many “morphospecies” designations that have only recently been resolved for western halictids (Gardner & Gibbs, 2020). Issues around identification of specimens, especially halictids, were also noted by Jamieson et al. (2019) in Colorado. In our prairie sites, several *Sphecodes* were either identified to morphospecies or to two species that could not be distinguished, *Lasioglossum trigeminum* and *L*. *callidum* could not be distinguished, and *Agapostemon texanus* and *A*. *angelicus* females could not be distinguished; an unknown number of *Lasioglossum* species were identified to “*tegulare* group.” The *Sphecodes* were mainly netted, but the *Lasioglossum* and *Agapostemon* species were most often trapped.

Bees captured by netting tend to be positively associated with floral abundance, while those captured by trapping often have a negative relationship (Kuhlman et al., 2021; O'Connor et al., 2019). Cane et al. (2000) suggested that flowers were more attractive than bowl traps to bees, resulting in lower capture rates when flowers were abundant. Observations by Pei et al. (2022) across 32 sites in North Dakota, USA, indicated fewer bees captured by bowl traps when flowers were abundant, while the reverse was true for netted captures. We found some support for this idea: At the wheatgrass sites, species richness and Shannon diversity of trapped bees were weakly negatively correlated with flower species richness, but not with any other measure of floral abundance or diversity; this contrasts with a lack of correlation between any measure of flower abundance or diversity with netted bee abundance or diversity in these sites. At the tallgrass sites, we found several positive correlations between netted bee abundance and diversity and forb abundance and diversity, but none for the trapped bees. Both of these sets of observations point toward different effects of floral abundance on captures by netting or trapping and lead us to concur with Rhoades et al. (2017) that comparing habitat associations as determined by different collection methods is problematic.

## 5 CONCLUSIONS

In summary, both netting and trapping have a place when designing studies involving bee diversity (Packer & Darla‐West, 2021), but challenges lie in distribution of effort between the two methods and interpretation of results within the ecological context where sampling occurs. As is true in any research project, the goals and objectives, as well as available resources, will dictate the amount and kind of data collected. Our results indicate that placing relatively more emphasis on netting and less on trapping, especially as emphasis on rare species declines (i.e., *q* increases), can lead to more complete estimates of bee diversity and simultaneously reduce the total number of bees captured, killed, and pinned for identification. An important benefit of capturing fewer individuals is reducing the effect of the study on an already crippling taxonomic bottleneck (Portman & Tepedino, 2021; Tepedino & Portman, 2021). If those who collect bees for scientific research were to include plots of number of species as a function of number of individuals captured in their workflow, the accumulation of data specific to different habitats would provide valuable benchmarks for optimizing sample sizes.

A separate, but important, consideration is comparability of sampling among all sites within a study, and this may be harder to achieve with netting than with bowl traps (Westphal et al., 2008). Nonetheless, time spent in training field personnel may ultimately be less costly than the extra time needed for processing and identifying greater numbers of bees captured in bowl traps. In any event, if both netting and trapping are required for a more complete species list, personnel will need to be trained to use nets.

Beyond comparability within a study, research on bee abundance and diversity is increasingly being used in meta‐analyses (Montero‐Castano & Vilá, 2012; Tonietto & Larkin, 2018), in which assumptions must be made about the comparability of estimates across studies. Given the variation in relationships between floral resources and bees as determined by netting versus trapping, combining these into a single meta‐analysis may be unwise if the goal is to make inferences about effects of habitat characteristics on bee diversity.

## AUTHOR CONTRIBUTIONS

**Diane L. Larson:** Conceptualization (equal); formal analysis (lead); methodology (lead); writing – original draft (lead). **Nora Pennarola:** Conceptualization (equal); data curation (equal); methodology (equal); writing – review and editing (equal). **Julia B. Leone:** Conceptualization (equal); data curation (equal); writing – review and editing (equal). **Jennifer L. Larson:** Conceptualization (equal); data curation (lead); writing – review and editing (equal).

## Supporting information


Data S1.

## Data Availability

Data for the wheatgrass sites can be found at Larson et al. (2018) and Larson and Larson (2023) and for the tallgrass sites at Leone et al. (2022a) Summaries of the data used in the analyses can be found in Tables A1 and A2. Code used for the analyses is in Supporting Information.

## APPENDIX A

**TABLE A1:** Bee species captured at wheatgrass prairie sites in South Dakota, USA.

Family	Species	Net (*N*)	Netted % of total	Trap (*N*)	Trapped % of total
Andrenidae	*Andrena helianthiformis*	6		0	
Andrenidae	*Andrena wilmattae*	3		0	
Andrenidae	*Calliopsis andreniformis*	1		8	
Andrenidae	*Perdita albipennis*	1		0	
Andrenidae	*Pseudopanurgus albitarsis*	2		0	
**Andrenidae**		**13**	1.00	**8**	0.11
Apidae	*Anthophora occidentalis*	3		0	
Apidae	*Anthophora walshii*	2		12	
Apidae	*Apis mellifera*	9		0	
Apidae	*Bombus auricomus*	0		1	
Apidae	*Bombus fervidus*	47		82	
Apidae	*Bombus griseocollis*	26		26	
Apidae	*Bombus huntii*	10		17	
Apidae	*Bombus morrisoni*	1		0	
Apidae	*Bombus nevadensis*	8		4	
Apidae	*Bombus pensylvanicus*	4		3	
Apidae	*Bombus rufocinctus*	3		2	
Apidae	*Diadasia BADL100*	8		0	
Apidae	*Epeolus bifasciatus*	1		0	
Apidae	*Eucera hamata*	0		1	
Apidae	*Melissodes agilis*	1		7	
Apidae	*Melissodes coloradensis*	0		2	
Apidae	*Melissodes communis*	7		24	
Apidae	*Melissodes coreopsis*	42		83	
Apidae	*Melissodes menuachus*	3		0	
Apidae	*Melissodes trinodis*	0		9	
Apidae	*Melitoma grisella*	1		10	
Apidae	*Svastra obliqua*	2		0	
Apidae	*Triepeolus BADL100*	1		0	
**Apidae**		**179**	13.74	**283**	3.82
Colletidae	*Colletes kincaidii*	2		1	
Colletidae	*Colletes latitarsis*	6		0	
Colletidae	*Colletes phaceliae*	0		1	
Colletidae	*Colletes robertsonii*	3		0	
Colletidae	*Hylaeus leptocephalus*	1		0	
**Colletidae**		**12**	0.92	**2**	0.03
Halictidae	*Agapostemon splendens*	0		1	
Halictidae	*Agapostemon texanus/angelicus*	6		128	
Halictidae	*Agapostemon virescens*	27		357	
Halictidae	*Augochlorella aurata*	154		1025	
Halictidae	*Augochloropsis metallica*	0		1	
Halictidae	*Halictus confusus*	26		188	
Halictidae	*Halictus ligatus*	7		40	
Halictidae	*Halictus parallelus*	2		2	
Halictidae	*Halictus rubicundus*	3		3	
Halictidae	*Halictus tripartitus*	20		282	
Halictidae	*Hoplitis pilosifrons*	2		4	
Halictidae	*Lasioglossum albipenne*	162		613	
Halictidae	*Lasioglossum ephialtum*	1		0	
Halictidae	*Lasioglossum incompletum*	3		8	
Halictidae	*Lasioglossum lusorium*	1		0	
Halictidae	*Lasioglossum nymphaearum*	1		0	
Halictidae	*Lasioglossum occidentale*	3		48	
Halictidae	*Lasioglossum packeri*	23		106	
Halictidae	*Lasioglossum paraforbesii*	2		0	
Halictidae	*Lasioglossum pectorale*	0		1	
Halictidae	*Lasioglossum pruinosum*	281		1705	
Halictidae	*Lasioglossum semicaeruleum*	117		1176	
Halictidae	*Lasioglossum simplex*	0		10	
Halictidae	*Lasioglossum trigeminum/callidum*	162		1356	
Halictidae	*Lasioglossum zephyrum*	0		1	
Halictidae	*Nomia universitatis*	2		0	
Halictidae	*Sphecodes BADL100*	6		0	
Halictidae	*Sphecodes BADL101*	1		0	
**Halictidae**		**1012**	77.67	**7055**	95.11
Megachilidae	*Anthidium porterae*	0		2	
Megachilidae	*Coelioxys BADL100*	1		0	
Megachilidae	*Dianthidium BADL100*	1		0	
Megachilidae	*Dianthidium concinnum*	3		29	
Megachilidae	*Dianthidium curvatum*	1		2	
Megachilidae	*Dianthidium sp_parvum1*	0		3	
Megachilidae	*Megachile brevis*	71		33	
Megachilidae	*Megachile parallela*	5		1	
Megachilidae	*Megachile pugnata*	1		0	
Megachilidae	*Osmia texana*	4		0	
**Megachilidae**		**87**	6.68	**70**	0.94
**Total captures**		**1303**		**7418**	

Bold values indicate the family and the values are the summaries for all species within that family.

**TABLE A2:** Bee species captured at tallgrass prairie sites in Minnesota, USA.

Family	Species	Net (*N*)	Netted % of total	Trap (*N*)	Trapped % of total
Andrenidae	*Andrena bisalicis*	0		1	
Andrenidae	*Andrena carlini*	1		9	
Andrenidae	*Andrena ceanothi*	3		2	
Andrenidae	*Andrena chromotricha*	2		0	
Andrenidae	*Andrena commoda*	1		61	
Andrenidae	*Andrena cressonii*	1		0	
Andrenidae	*Andrena erythrogaster*	0		1	
Andrenidae	*Andrena forbesii*	1		1	
Andrenidae	*Andrena helianthi*	2		0	
Andrenidae	*Andrena helianthiformis*	1		0	
Andrenidae	*Andrena hirticincta*	1		0	
Andrenidae	*Andrena milwaukeensis*	0		2	
Andrenidae	*Andrena nivalis*	0		1	
Andrenidae	*Andrena placata*	1		0	
Andrenidae	*Andrena simplex*	0		1	
Andrenidae	*Andrena thaspii*	0		17	
Andrenidae	*Andrena wilkella*	11		3	
Andrenidae	*Andrena ziziae*	8		2	
Andrenidae	*Calliopsis nebraskensis*	1		0	
Andrenidae	*Perdita perpallida*	2		0	
Andrenidae	*Perdita swenki*	1		10	
Andrenidae	*Protandrena bancrofti*	1		3	
**Andrenidae**		**38**	6.92	**114**	0.97
Apidae	*Anthophora terminalis*	0		3	
Apidae	*Anthophora walshii*	2		0	
Apidae	*Apis mellifera*	95		219	
Apidae	*Bombus auricomus*	6		6	
Apidae	*Bombus bimaculatus*	4		6	
Apidae	*Bombus borealis*	9		9	
Apidae	*Bombus fervidus*	7		31	
Apidae	*Bombus griseocollis*	36		20	
Apidae	*Bombus impatiens*	14		3	
Apidae	*Bombus pensylvanicus*	4		11	
Apidae	*Bombus rufocinctus*	1		0	
Apidae	*Bombus ternarius*	2		2	
Apidae	*Bombus terricola*	1		0	
Apidae	*Bombus vagans*	13		5	
Apidae	*Ceratina dupla*	0		2	
Apidae	*Ceratina mikmaqi*	20		120	
Apidae	*Eucera hamata*	0		17	
Apidae	*Melissodes agilis*	4		16	
Apidae	*Melissodes bimaculatus*	0		19	
Apidae	*Melissodes communis*	0		1	
Apidae	*Melissodes denticulatus*	0		3	
Apidae	*Melissodes desponsus*	11		14	
Apidae	*Melissodes druriellus*	1		2	
Apidae	*Melissodes trinodis*	79		319	
Apidae	*Nomada articulata*	0		1	
Apidae	*Nomada near_MR_2*	1		0	
Apidae	*Svastra obliqua*	1		0	
Apidae	*Triepeolus donatus*	0		2	
Apidae	*Xenoglossa kansensis*	0		6	
**Apidae**		**311**	56.65	**837**	7.10
Colletidae	*Colletes kincaidii*	1		5	
Colletidae	*Colletes robertsonii*	1		0	
Colletidae	*Colletes simulans*	1		1	
Colletidae	*Colletes solidaginis*	1		1	
Colletidae	*Colletes susannae*	1		0	
Colletidae	*Hylaeus affinis*	12		51	
Colletidae	*Hylaeus mesillae*	1		8	
Colletidae	*Hylaeus nelumbonis*	1		1	
**Colletidae**		**19**	3.46	**67**	0.57
Halictidae	*Agapostemon sericeus*	0		5	
Halictidae	*Agapostemon texanus*	5		306	
Halictidae	*Agapostemon virescens*	6		987	
Halictidae	*Augochlorella aurata*	66		1232	
Halictidae	*Augochloropsis metallica*	0		4	
Halictidae	*Halictus confusus*	15		303	
Halictidae	*Halictus ligatus*	19		85	
Halictidae	*Halictus parallelus*	0		37	
Halictidae	*Halictus rubicundus*	0		21	
Halictidae	*Lasioglossum “tegulare group”*	1		126	
Halictidae	*Lasioglossum admirandum*	20		678	
Halictidae	*Lasioglossum albipenne*	16		1575	
Halictidae	*Lasioglossum cattellae*	0		1	
Halictidae	*Lasioglossum cinctipes*	0		6	
Halictidae	*Lasioglossum coriaceum*	2		377	
Halictidae	*Lasioglossum cressonii*	1		51	
Halictidae	*Lasioglossum ephialtum*	1		114	
Halictidae	*Lasioglossum foxii*	0		1	
Halictidae	*Lasioglossum hitchensi*	0		6	
Halictidae	*Lasioglossum imitatum*	0		1	
Halictidae	*Lasioglossum laevissimum*	2		10	
Halictidae	*Lasioglossum leucocomum*	2		117	
Halictidae	*Lasioglossum leucozonium*	1		22	
Halictidae	*Lasioglossum lineatulum*	0		16	
Halictidae	*Lasioglossum michiganense*	0		3	
Halictidae	*Lasioglossum novascotiae*	0		421	
Halictidae	*Lasioglossum paradmirandum*	0		3	
Halictidae	*Lasioglossum paraforbesii*	6		175	
Halictidae	*Lasioglossum pectorale*	1		9	
Halictidae	*Lasioglossum perpunctatum*	1		5	
Halictidae	*Lasioglossum pilosum*	0		3	
Halictidae	*Lasioglossum planatum*	0		6	
Halictidae	*Lasioglossum pruinosum*	13		2370	
Halictidae	*Lasioglossum semicaeruleum*	1		248	
Halictidae	*Lasioglossum subviridatum*	0		1	
Halictidae	*Lasioglossum truncatum*	0		2	
Halictidae	*Lasioglossum versans*	0		17	
Halictidae	*Lasioglossum versatum*	0		1379	
Halictidae	*Lasioglossum vierecki*	0		3	
Halictidae	*Lasioglossum viridatum*	0		6	
Halictidae	*Lasioglossum weemsi*	0		8	
Halictidae	*Lasioglossum zephyrum*	0		7	
Halictidae	*Lasioglossum zonulum*	0		16	
Halictidae	*Nomia universitatis*	2		0	
Halictidae	*Sphecodes atlantis/cressonii*	0		1	
Halictidae	*Sphecodes davisii*	0		2	
Halictidae	*Sphecodes mandibubris*	0		1	
**Halictidae**		**181**	32.97	**10,767**	91.35
Megachilidae	*Coelioxys octodentata*	0		1	
Megachilidae	*Coelioxys rufitarsis*	0		1	
Megachilidae	*Dianthidium simile*	0		2	
Megachilidae	*Heriades carinata*	1		1	
Megachilidae	*Heriades leavitti*	3		0	
Megachilidae	*Hoplitis pilosifrons*	2		82	
Megachilidae	*Megachile brevis*	0		2	
Megachilidae	*Megachile latimanus*	15		16	
Megachilidae	*Megachile mendica*	1		1	
Megachilidae	*Megachile montivaga*	0		4	
Megachilidae	*Megachile relativa*	1		1	
Megachilidae	*Osmia near_collinsiae*	0		3	
Megachilidae	*Stelis lateralis*	0		2	
**Megachilidae**		**23**	4.19	**116**	0.98
**Total captures**		**549**		**11,787**	

Bold values indicate the family and the values are the summaries for all species within that family.
