# Supplementary material for: Fewer bowl traps and more hand netting can increase effective number of bee species and reduce excessive captures
Source: Ecol Evol. 2024 Feb 26;14(2):e11036. doi: 10.1002/ece3.11036 (PMC10897529; doi:10.1002/ece3.11036)
Supplement: Supplementary file 1 — Data S1. [file ECE3-14-e11036-s001.docx]

Code used in “A comparison of effective number of bee species captured by hand netting and bowl trapping.”

Three R Markdown documents and one short SAS program are included here. The R code is written to access the wheatgrass prairie data, but the same code works for both of the data sets. The analysis that created the data submitted to SAS was done with PCOrd, which is menu-driven, so there is no code for that analysis.

Mixed-grass sites diversity comparison

Diane Larson

2023-03-10

## R Markdown

This is an R Markdown document to compare Hill diversity profiles for netted and trapped samples.

library(iNEXT)
library(ggplot2)
library(openxlsx)
library(SpadeR)

setwd("C:\\Users\\dlarson\\Documents\\bee bowls v nets")

Netted diversity profile.

blnet1 <- read.xlsx('C:\\Users\\dlarson\\Documents\\bee bowls v nets\\netvtrapall.xlsx', sheet = "net")

netout <- Diversity(blnet1, datatype = c("abundance"), q = NULL)

netout

##
## (1) BASIC DATA INFORMATION:
## Variable Value
## Sample size n 1303
## Number of observed species D 59
## Estimated sample coverage C 0.988
## Estimated CV CV 2.313
##
## (2) ESTIMATION OF SPECIES RICHNESS (DIVERSITY OF ORDER 0):
##
## Estimate s.e. 95%Lower 95%Upper
## Chao1 (Chao, 1984) 75.0 10.6 63.9 111.1
## Chao1-bc 72.3 8.8 63.1 102.5
## iChao1 75.0 10.6 63.9 111.1
## ACE (Chao & Lee, 1992) 73.1 7.2 64.5 95.3
## ACE-1 (Chao & Lee, 1992) 77.4 10.4 65.5 110.7
##
## Descriptions of richness estimators (See Species Part)
##
## (3a) SHANNON ENTROPY:
##
## Estimate s.e. 95%Lower 95%Upper
## MLE 2.719 0.039 2.643 2.795
## Jackknife 2.749 0.040 2.671 2.827
## Chao & Shen 2.770 0.041 2.691 2.849
## Chao et al. (2013) 2.750 0.039 2.673 2.827
##
## MLE: empirical or observed entropy.
## Jackknife: see Zahl (1977).
## Chao & Shen: based on the Horvitz-Thompson estimator and sample coverage method; see Chao and Shen (2003).
## see Chao and Shen (2003).
## Chao et al. (2013): A nearly optimal estimator of Shannon entropy; see Chao et al. (2013).
## Estimated standard error is computed based on a bootstrap method.
##
## (3b) SHANNON DIVERSITY (EXPONENTIAL OF SHANNON ENTROPY):
##
## Estimate s.e. 95%Lower 95%Upper
## MLE 15.165 0.574 14.039 16.290
## Jackknife 15.625 0.603 14.443 16.807
## Chao & Shen 15.960 0.627 14.730 17.189
## Chao et al. (2013) 15.644 0.598 14.473 16.815
##
## (4a) SIMPSON CONCENTRATION INDEX:
##
## Estimate s.e. 95%Lower 95%Upper
## MVUE 0.10628 0.00367 0.09909 0.11348
## MLE 0.10697 0.00367 0.09978 0.11416
##
## MVUE: minimum variance unbiased estimator; see Eq. (2.27) of Magurran (1988).
## MLE: maximum likelihood estimator or empirical index; see Eq. (2.26) of Magurran (1988).
##
## (4b) SIMPSON DIVERSITY (INVERSE OF SIMPSON CONCENTRATION):
##
## Estimate s.e. 95%Lower 95%Upper
## MVUE 9.40872 0.32189 8.77783 10.03962
## MLE 9.34840 0.31756 8.72599 9.97081
##
## (5) CHAO AND JOST (2015) ESTIMATES OF HILL NUMBERS
##
## q ChaoJost 95%Lower 95%Upper Empirical 95%Lower 95%Upper
## 1 0.00 74.988 51.221 98.755 59.000 53.071 64.929
## 2 0.25 47.761 38.880 56.642 39.842 36.136 43.548
## 3 0.50 30.409 27.361 33.457 27.101 24.798 29.404
## 4 0.75 20.748 19.162 22.334 19.498 17.963 21.033
## 5 1.00 15.644 14.486 16.802 15.165 14.034 16.296
## 6 1.25 12.854 11.921 13.787 12.646 11.735 13.557
## 7 1.50 11.206 10.402 12.010 11.094 10.306 11.882
## 8 1.75 10.145 9.416 10.874 10.070 9.353 10.787
## 9 2.00 9.409 8.721 10.097 9.348 8.670 10.026
## 10 2.25 8.866 8.200 9.532 8.812 8.153 9.471
## 11 2.50 8.445 7.786 9.104 8.394 7.745 9.043
## 12 2.75 8.107 7.450 8.764 8.058 7.409 8.707
## 13 3.00 7.828 7.167 8.489 7.779 7.128 8.430
##
## ChaoJost: diversity profile estimator derived by Chao and Jost (2015).
## Empirical: maximum likelihood estimator (observed index).
##


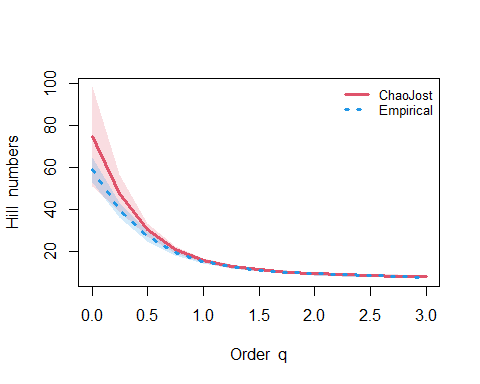


Trapped diversity profile.

bltrap1 <- read.xlsx('C:\\Users\\dlarson\\Documents\\bee bowls v nets\\netvtrapall.xlsx', sheet = "trap")

trapout <- Diversity(bltrap1, datatype = c("abundance"), q = NULL)

trapout

##
## (1) BASIC DATA INFORMATION:
## Variable Value
## Sample size n 7418
## Number of observed species D 45
## Estimated sample coverage C 0.999
## Estimated CV CV 2.328
##
## (2) ESTIMATION OF SPECIES RICHNESS (DIVERSITY OF ORDER 0):
##
## Estimate s.e. 95%Lower 95%Upper
## Chao1 (Chao, 1984) 53.1 7.1 46.8 80.6
## Chao1-bc 51.0 5.4 46.3 72.1
## iChao1 55.1 4.7 49.2 69.1
## ACE (Chao & Lee, 1992) 54.5 6.3 47.9 76.1
## ACE-1 (Chao & Lee, 1992) 58.5 10.1 48.7 95.0
##
## Descriptions of richness estimators (See Species Part)
##
## (3a) SHANNON ENTROPY:
##
## Estimate s.e. 95%Lower 95%Upper
## MLE 2.270 0.013 2.244 2.297
## Jackknife 2.274 0.013 2.248 2.300
## Chao & Shen 2.278 0.013 2.251 2.304
## Chao et al. (2013) 2.274 0.013 2.248 2.301
##
## MLE: empirical or observed entropy.
## Jackknife: see Zahl (1977).
## Chao & Shen: based on the Horvitz-Thompson estimator and sample coverage method; see Chao and Shen (2003).
## see Chao and Shen (2003).
## Chao et al. (2013): A nearly optimal estimator of Shannon entropy; see Chao et al. (2013).
## Estimated standard error is computed based on a bootstrap method.
##
## (3b) SHANNON DIVERSITY (EXPONENTIAL OF SHANNON ENTROPY):
##
## Estimate s.e. 95%Lower 95%Upper
## MLE 9.683 0.130 9.428 9.938
## Jackknife 9.719 0.131 9.463 9.975
## Chao & Shen 9.753 0.131 9.496 10.009
## Chao et al. (2013) 9.720 0.131 9.463 9.976
##
## (4a) SIMPSON CONCENTRATION INDEX:
##
## Estimate s.e. 95%Lower 95%Upper
## MVUE 0.14248 0.00156 0.13943 0.14554
## MLE 0.14260 0.00156 0.13955 0.14565
##
## MVUE: minimum variance unbiased estimator; see Eq. (2.27) of Magurran (1988).
## MLE: maximum likelihood estimator or empirical index; see Eq. (2.26) of Magurran (1988).
##
## (4b) SIMPSON DIVERSITY (INVERSE OF SIMPSON CONCENTRATION):
##
## Estimate s.e. 95%Lower 95%Upper
## MVUE 7.01837 0.08988 6.84221 7.19453
## MLE 7.01268 0.08972 6.83683 7.18853
##
## (5) CHAO AND JOST (2015) ESTIMATES OF HILL NUMBERS
##
## q ChaoJost 95%Lower 95%Upper Empirical 95%Lower 95%Upper
## 1 0.00 53.099 26.549 79.649 45.000 41.345 48.655
## 2 0.25 27.415 22.297 32.533 25.065 23.779 26.351
## 3 0.50 16.443 15.608 17.278 15.880 15.388 16.372
## 4 0.75 11.863 11.551 12.175 11.731 11.429 12.033
## 5 1.00 9.720 9.467 9.973 9.683 9.430 9.936
## 6 1.25 8.557 8.326 8.788 8.543 8.312 8.774
## 7 1.50 7.843 7.623 8.063 7.835 7.615 8.055
## 8 1.75 7.363 7.151 7.575 7.357 7.147 7.567
## 9 2.00 7.018 6.812 7.224 7.013 6.807 7.219
## 10 2.25 6.758 6.556 6.960 6.753 6.551 6.955
## 11 2.50 6.554 6.352 6.756 6.549 6.349 6.749
## 12 2.75 6.389 6.189 6.589 6.383 6.183 6.583
## 13 3.00 6.252 6.050 6.454 6.246 6.044 6.448
##
## ChaoJost: diversity profile estimator derived by Chao and Jost (2015).
## Empirical: maximum likelihood estimator (observed index).
##


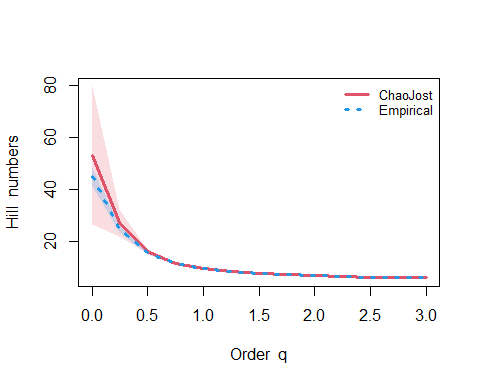


Combined netted and trapped

blboth <- read.xlsx('C:\\Users\\dlarson\\Documents\\bee bowls v nets\\netvtrapall.xlsx', sheet = "combined")

bothout <- Diversity(blboth, datatype = c("abundance"), q = NULL)

bothout

##
## (1) BASIC DATA INFORMATION:
## Variable Value
## Sample size n 8721
## Number of observed species D 71
## Estimated sample coverage C 0.998
## Estimated CV CV 2.935
##
## (2) ESTIMATION OF SPECIES RICHNESS (DIVERSITY OF ORDER 0):
##
## Estimate s.e. 95%Lower 95%Upper
## Chao1 (Chao, 1984) 101.1 19.3 80.5 165.9
## Chao1-bc 95.4 15.2 79.0 145.8
## iChao1 108.5 12.3 91.1 141.0
## ACE (Chao & Lee, 1992) 89.5 8.9 78.5 116.4
## ACE-1 (Chao & Lee, 1992) 96.9 14.2 80.5 141.6
##
## Descriptions of richness estimators (See Species Part)
##
## (3a) SHANNON ENTROPY:
##
## Estimate s.e. 95%Lower 95%Upper
## MLE 2.380 0.012 2.356 2.404
## Jackknife 2.386 0.012 2.361 2.410
## Chao & Shen 2.392 0.012 2.367 2.416
## Chao et al. (2013) 2.386 0.012 2.362 2.411
##
## MLE: empirical or observed entropy.
## Jackknife: see Zahl (1977).
## Chao & Shen: based on the Horvitz-Thompson estimator and sample coverage method; see Chao and Shen (2003).
## see Chao and Shen (2003).
## Chao et al. (2013): A nearly optimal estimator of Shannon entropy; see Chao et al. (2013).
## Estimated standard error is computed based on a bootstrap method.
##
## (3b) SHANNON DIVERSITY (EXPONENTIAL OF SHANNON ENTROPY):
##
## Estimate s.e. 95%Lower 95%Upper
## MLE 10.808 0.133 10.548 11.069
## Jackknife 10.866 0.134 10.603 11.129
## Chao & Shen 10.934 0.136 10.668 11.201
## Chao et al. (2013) 10.874 0.134 10.611 11.136
##
## (4a) SIMPSON CONCENTRATION INDEX:
##
## Estimate s.e. 95%Lower 95%Upper
## MVUE 0.13513 0.00154 0.13212 0.13814
## MLE 0.13523 0.00154 0.13222 0.13824
##
## MVUE: minimum variance unbiased estimator; see Eq. (2.27) of Magurran (1988).
## MLE: maximum likelihood estimator or empirical index; see Eq. (2.26) of Magurran (1988).
##
## (4b) SIMPSON DIVERSITY (INVERSE OF SIMPSON CONCENTRATION):
##
## Estimate s.e. 95%Lower 95%Upper
## MVUE 7.40035 0.07335 7.25658 7.54411
## MLE 7.39492 0.07323 7.25138 7.53846
##
## (5) CHAO AND JOST (2015) ESTIMATES OF HILL NUMBERS
##
## q ChaoJost 95%Lower 95%Upper Empirical 95%Lower 95%Upper
## 1 0.00 101.080 65.698 136.462 71.000 64.260 77.740
## 2 0.25 44.189 36.080 52.298 36.197 33.821 38.573
## 3 0.50 21.977 20.527 23.427 20.363 19.573 21.153
## 4 0.75 14.064 13.699 14.429 13.760 13.441 14.079
## 5 1.00 10.874 10.684 11.064 10.808 10.622 10.994
## 6 1.25 9.307 9.158 9.456 9.288 9.141 9.435
## 7 1.50 8.401 8.266 8.536 8.392 8.257 8.527
## 8 1.75 7.813 7.682 7.944 7.807 7.676 7.938
## 9 2.00 7.400 7.269 7.531 7.395 7.264 7.526
## 10 2.25 7.093 6.962 7.224 7.088 6.957 7.219
## 11 2.50 6.855 6.724 6.986 6.850 6.719 6.981
## 12 2.75 6.663 6.530 6.796 6.658 6.525 6.791
## 13 3.00 6.505 6.370 6.640 6.500 6.365 6.635
##
## ChaoJost: diversity profile estimator derived by Chao and Jost (2015).
## Empirical: maximum likelihood estimator (observed index).
##


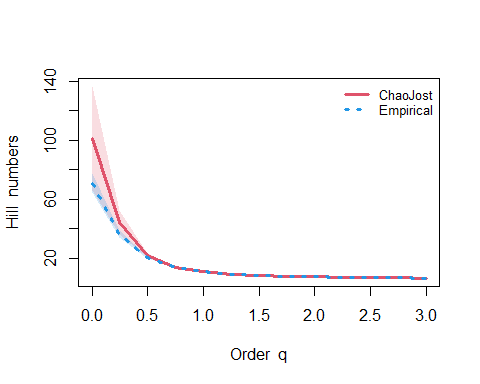


Estimated number of shared species between netted and trapped samples.

blall1 <- read.xlsx('C:\\Users\\dlarson\\Documents\\bee bowls v nets\\netvtrapall.xlsx', sheet = "all")

blshared <- ChaoShared(blall1, datatype = c("abundance"), se = TRUE, nboot = 200, conf = 0.95)

blshared

##
## (1) BASIC DATA INFORMATION:
##
## Sample size in Community 1 n1 = 1303
## Sample size in Community 2 n2 = 7418
## Number of observed species in Community 1 D1 = 59
## Number of observed species in Community 2 D2 = 45
## Number of observed shared species D12 = 33
## Bootstrap replications for s.e. estimate 200
##
## "Entire" Shared Species Group:
## Some statistics:
## ---------------------------------------------------------------------------
## f[11] = 0 ; f[1+] = 4 ; f[+1] = 2 ; f[2+] = 4 ; f[+2] = 3 ; f[22] = 1
## ---------------------------------------------------------------------------
##
## "Rare" Shared Species Group: (Both frequencies can only up to 10)
## Some statistics:
## -------------------------------------------------------------------
## f[1+]_rare = 4 ; f[+1]_rare = 2 ; f[2+]_rare = 3 ; f[+2]_rare = 3
## -------------------------------------------------------------------
## Number of observed individuals in Community 1 n1_rare = 107
## Number of observed individuals in Community 2 n2_rare = 88
## Number of observed shared species D12_rare = 13
## Estimated sample coverage C12_rare = 0.736
## Estimated CCVs CCV_1 = 0.427
## CCV_2 = 0.334
## CCV_12 = -0.471
##
##
## (2) ESTIMATION RESULTS OF THE NUMBER OF SHARED SPECIES:
##
## Estimate s.e. 95%Lower 95%Upper
## Homogeneous 37.653 4.122 34.047 53.680
## Heterogeneous(ACE-shared) 40.881 9.449 34.239 83.123
## Chao1-shared 35.665 2.868 33.478 47.870
## Chao1-shared-bc 34.449 1.915 33.202 43.388
##
##
## (3) DESCRIPTION OF MODELS FOR ESTIMATING SHARED SPECIES RICHNESS:
##
## Homogeneous: This model assumes that the shared species in each community have the same discovery probabilities; see the Eq. (3.11a) of Chao et al. (2000).
##
## Heterogeneous (ACE-shared): This model allows for heterogeneous discovery probabilities among shared species; see Eq. (3.11b) of Chao et al. (2000). It is an extension of the ACE estimator to two communities. It is replaced by Chao1-shared when the estimated sample coverage for rare shared species group (C12_rare in the output) is zero.
##
## Chao1-shared: An extension of the Chao1 estimator to estimate shared species richness between two communities. It provides a lower bound of shared species richness. See Eq. (3.6) of Pan et al. (2009). It is replaced by Chao1-shared-bc for the case f[2+]=0 or f[+2]=0.
##
## Chao1-shared-bc: A bias-corrected form of Chao1-shared estimator; See Pan et al. (2009).
##

End.

Diversity comparison: Nets v traps for mixed grass prairie sites

Diane Larson

2023-08-02

## R Markdown

This set of analyses compares net v trap captures for all captures, and within families for the mixed-grass prairie sites. Andrenids and Colletids did not have enough captures for this analysis.

library(iNEXT)

## Warning: package 'iNEXT' was built under R version 4.2.2

library(ggplot2)

## Warning: package 'ggplot2' was built under R version 4.2.3

library(openxlsx)
library(SpadeR)

setwd("C:\\Users\\dlarson\\Documents\\bee bowls v nets")

Badlands all

blall <- read.xlsx('C:\\Users\\dlarson\\Documents\\bee bowls v nets\\netvtrapall.xlsx', sheet = "all")

blallout <- iNEXT(blall, q=c(0,1,2), datatype="abundance", size=NULL, endpoint=NULL, knots=40,
 se=TRUE, conf=0.95, nboot=50)

ggiNEXT(blallout, type=1, se=TRUE, facet.var="Order.q", color.var="Assemblage", grey=FALSE)


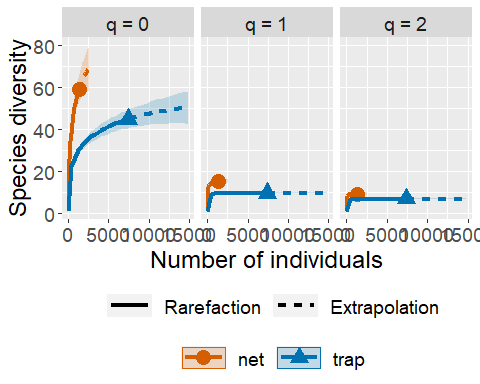


write.csv(blallout[["iNextEst"]][["size_based"]], file="blall.csv", row.names = FALSE)

Two-assemblage similarity measures from SpadeR

blallsim <- SimilarityPair(blall, datatype = c("abundance"), nboot = 200)

blallsim

## (1) BASIC DATA INFORMATION:
##
## The loaded set includes abundance/incidence data from 2 communities
## and a total of 71 species.
##
## Samples size in Community 1 n1 = 1303
## Samples size in Community 2 n2 = 7418
## Number of observed species in Community 1 D1 = 59
## Number of observed species in Community 2 D2 = 45
## Number of observed shared species in two communities D12 = 33
## Number of bootstrap replications for s.e. estimate 200
##
## Some statistics:
## f[11]= 0 ; f[1+]= 4 ; f[+1]= 2 ; f[2+]= 4 ; f[+2]= 3 ; f[22]= 1
##
## (2) EMPIRICAL SIMILARITY INDICES:
##
## Estimate s.e. 95%Lower 95%Upper
## (a) Classic richness-based similarity
##
## C02 (q=0, Sorensen) 0.6346 0.0302 0.5754 0.6938
## U02 (q=0, Jaccard) 0.4648 0.0300 0.4060 0.5236
##
## (b) Measures for comparing species relative abundances
##
## C12=U12 (q=1, Horn) 0.9042 0.0068 0.8908 0.9177
##
## C22 (q=2, Morisita-Horn) 0.9354 0.0079 0.9199 0.9510
## U22 (q=2, Regional overlap) 0.9666 0.0043 0.9583 0.9750
##
## ChaoJaccard-abundance 0.9415 0.0082 0.9254 0.9576
## ChaoSorensen-abundance 0.9699 0.0044 0.9613 0.9785
##
## (c) Measures for comparing size-weighted species relative abundances
##
## Horn size-weighted (q=1) 0.8982 0.0079 0.8828 0.9136
##
## (d) Measures for comparing species absolute abundances
##
## C12=U12 (q=1) 0.5464 0.0079 0.5310 0.5618
##
## C22 (Morisita-Horn) 0.2811 0.0054 0.2704 0.2917
## U22 (Regional overlap) 0.4388 0.0066 0.4258 0.4518
##
## Bray-Curtis 0.2713 0.0032 0.2650 0.2776
##
## (3) ESTIMATED SIMILARITY INDICES:
##
## Estimate s.e. 95%Lower 95%Upper
## (a) Classic richness-based similarity:
##
## C02 (q=0, Sorensen) 0.5569 0.0769 0.4062 0.7076
## U02 (q=0, Jaccard) 0.3859 0.0733 0.2422 0.5296
##
## (b) Measures for comparing species relative abundances
##
## C12=U12 (q=1, Horn) 0.9061 0.0079 0.8906 0.9215
##
## C22 (q=2, Morisita-Horn) 0.9385 0.0080 0.9229 0.9540
## U22 (q=2, Regional overlap) 0.9683 0.0042 0.9599 0.9766
##
## ChaoJaccard-abundance 0.9449 0.0129 0.9196 0.9702
## ChaoSorensen-abundance 0.9717 0.0068 0.9584 0.9850
##
## (c) Measures for comparing size-weighted species relative abundances
##
## Horn size-weighted (q=1) 0.9000 0.0088 0.8828 0.9172
##
## (d) Measures for comparing species absolute abundances
##
## C12=U12 (q=1) 0.5475 0.0088 0.5304 0.5647
##
## C22 (q=2, Morisita-Horn) 0.2814 0.0054 0.2707 0.2921
## U22 (q=2, Regional overlap) 0.4392 0.0066 0.4262 0.4522
##
## Bray-Curtis 0.2722 0.0033 0.2657 0.2787
##
## NOTE: If an estimate is greater than 1, it is replaced by 1.

Apidae: netted sample size 155 individuals.

blapid <- read.xlsx('C:\\Users\\dlarson\\Documents\\bee bowls v nets\\netvtrapall.xlsx', sheet = "apid")

blapidout <- iNEXT(blapid, q=c(0,1,2), datatype="abundance", size=NULL, endpoint=NULL, knots=40,
 se=TRUE, conf=0.95, nboot=50)

ggiNEXT(blapidout, type=1, se=TRUE, facet.var="Order.q", color.var="Assemblage", grey=FALSE)


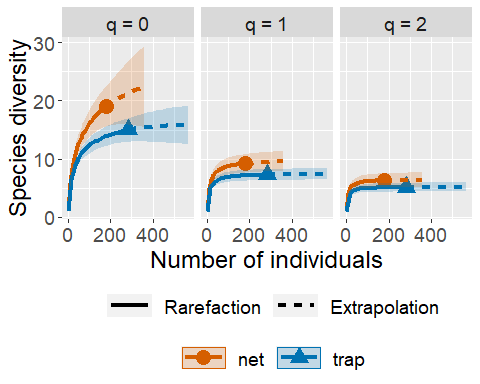


write.csv(blapidout[["iNextEst"]][["size_based"]], file="blapid.csv", row.names = FALSE)

Halictidae: netted sample size 1036 individuals.

blhalictid <- read.xlsx('C:\\Users\\dlarson\\Documents\\bee bowls v nets\\netvtrapall.xlsx', sheet = "halictid")

blhalout <- iNEXT(blhalictid, q=c(0,1,2), datatype="abundance", size=NULL, endpoint=NULL, knots=40,
 se=TRUE, conf=0.95, nboot=50)

ggiNEXT(blhalout, type=1, se=TRUE, facet.var="Order.q", color.var="Assemblage", grey=FALSE)


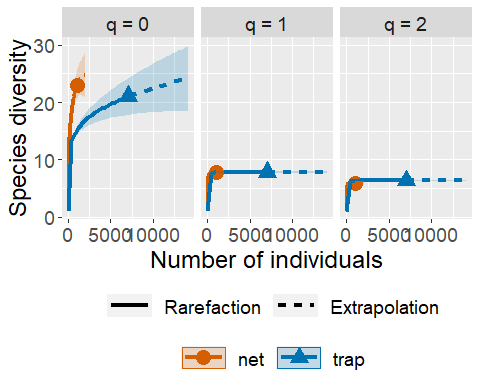


write.csv(blhalout[["iNextEst"]][["size_based"]], file="blhalictid.csv", row.names = FALSE)

Megachilidae: netted sample size 87 individuals.

blmega <- read.xlsx('C:\\Users\\dlarson\\Documents\\bee bowls v nets\\netvtrapall.xlsx', sheet = "megachilid")

blmegaout <- iNEXT(blmega, q=c(0,1,2), datatype="abundance", size=NULL, endpoint=NULL, knots=40,
 se=TRUE, conf=0.95, nboot=50)

ggiNEXT(blmegaout, type=1, se=TRUE, facet.var="Order.q", color.var="Assemblage", grey=FALSE)


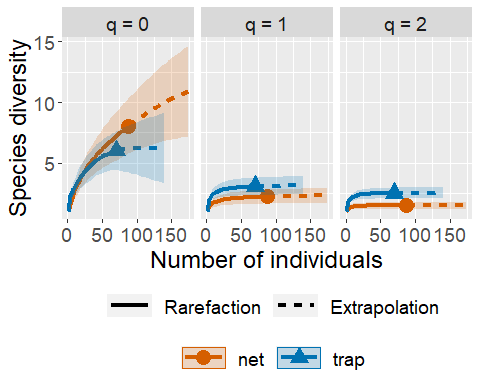


write.csv(blmegaout[["iNextEst"]][["size_based"]], file="blmegachilid.csv", row.names = FALSE)

End.

***SAS Code for correlations between bee captures and flower metrics at wheatgrass prairie sites;

LIBNAME EX XLSX 'C:\Users\dlarson\Documents\bee bowls v nets\pcord output\Badlands\flower and bee summary from pcord.xlsx';

**DATA** bees;

SET EX.flandbees;

**run**;

**proc** **corr** data=bees;

title 'net and trap corrs with flowers';

var Meannet netS netE netHprime netD Meantrap trapS trapE trapHprime trapD;

with Meanflct flS flE flHprime flD;

**run**;

***Code for correlations between bee captures and forb metrics at tallgrass prairie sites;

LIBNAME EX XLSX 'C:\Users\dlarson\Documents\bee bowls v nets\burn-graze data\bg bees&forbs.xlsx';

**Data** forbs;

set ex.toanalyze;

**run**;

**proc** **corr** data=forbs;

title 'all years';

var Xnetall Snetall Enetall Hprnetall Dnetall Xtrapall Strapall Etrapall Hprtrapall Dtrapall;

with MeanAll Sall Eall HprAll Dall;

**run**;
